# Quantification of node importance in rain gauge network: influence of temporal resolution and rain gauge density

**DOI:** 10.1038/s41598-020-66363-5

**Published:** 2020-06-17

**Authors:** Shubham Tiwari, Sanjeev Kumar Jha, Ankit Singh

**Affiliations:** Indian Institute of Science Education and Research, Bhopal, Madhya Pradesh India

**Keywords:** Environmental sciences, Hydrology

## Abstract

Rain gauge network is important for collecting rainfall information effectively and efficiently. Rain gauge networks have been studied for several decades from a range of hydrological perspectives, where rain gauges with unique or non-repeating information are considered as important. However, the problem of quantification of node importance and subsequent identification of the most important nodes in rain gauge networks have not yet been extensively addressed in the literature. In this study, we use the concept of the complex networks to evaluate the Indian Meteorological Department (IMD) monitored 692 rain gauge in the Ganga River Basin. We consider the complex network theory-based Degree Centrality (DC), Clustering Coefficient (CC) and Mutual Information (MI) as the parameters to quantify the rainfall variability associated with all the rain gauges in the network. Multiple rain gauge network scenario with varying rain gauge density (i.e. Network Size (NS) = 173, 344, 519, and 692) and Temporal Resolution (i.e. TR = 3 hours, 1 day, and 1 month) are introduced to study the effect of rain gauge density, gauge location and temporal resolution on the node importance quantification. Proxy validation of the methodology was done using a hydrological model. Our results indicate that the network density and temporal resolution strongly influence a node’s importance in rain gauge network. In addition, we concluded that the degree centrality along with clustering coefficient is the preferred parameter than the mutual information for the node importance quantification. Furthermore, we observed that the network properties (spatial distribution, DC, Collapse Correlation Threshold (CCT), CC Range distributions) associated with TR = 3 hours and 1 day are comparable whereas TR = 1 month exhibit completely different trends. We also found that the rain gauges situated at high elevated areas are extremely important irrespective of the NS and TR. The encouraging results for the quantification of nodes importance in this study seem to indicate that the approach has the potential to be used in extreme rainfall forecasting, in studying changing rainfall patterns and in filling gaps in spatial data. The technique can be further helpful in the ground-based observation network design of a wide range of meteorological parameters with spatial correlation.

## Introduction

Precise rainfall information at high spatial and temporal resolution are highly desirable in various research fields such as hydrological simulation, water resources management, flood forecasting etc^1–5^. Rainfall is a phenomenon marked by high variability both in space and time^6^, which makes its measurement difficult. A number of sophisticated approaches, such as weather radar, satellite rainfall estimation algorithms and numerical weather models^7^, may be employed to estimate the temporal and spatial characteristics of rainfall. Nevertheless, in order to minimise the measurement errors^8^, most methods require a calibration and validation process with the recorded rainfall data from the existing rain gauge networks^9^. Thus, reliable rain gauge networks are essential to provide a strong basis for the interpretation of the spatio-temporal characteristics of rainfall. For a given rain gauge network, there are several major challenges. First, the measurement of rainfall can suffer from systematic errors, random errors and gaps (missing data)^10^. Secondly, the network was likely built based solely on accessibility and available budget; for example, many rain gauges were installed near residential areas so that they could be operated and maintained conveniently; very few or no monitoring sites are in remote or mountainous areas; such configurations are not optimal for water resources management, flood forecasting, hydrological analysis etc^11^. Thirdly, high density of rain gauges is always desirable in a basin, however rarely found^12–15^ and hence there is no specific answer to the key question: what is the size of rain gauge network that is sufficient to record the spatio-temporal variability of the rainfall in a basin?

Given several uncertainties associated with the rain gauge networks, quantification of rain gauge importance in a network becomes significant. There are many different approaches for quantifying node importance (see Boccaletti^16^ for a general account of this topic). Borgatti^17^ used node importance quantification to identify the sets of key players in a social network. Liu *et al*.^18^ proposed node importance measurement based on the concept of degree centrality, betweenness centrality and closeness centrality. In graph/network theory, important node (critical node) means the node whose removal may result into maximum degradation of graph connectivity. Since in case of rain gauge network the structural connectivity of network is rather not very significant, rain gauges with non-repeating (unique) information are considered as important. There are several methods available for rain gauge network evaluation based on variance reduction^19–24^, dimension reduction^25–28^, kriging^29–31^, entropy^32–37^, optimization^38–40^, hybrid of several approaches^41–44^ (see Mishra and Coulibaly^45^ for a more detailed discussion on this topic). Dai^46^ proposed a scheme for rain gauge network design based on remotely sensed rainfall measurements. A frequently used measure in rain gauge network evaluation is mutual information (based on Shannon Entropy), which quantifies the amount of information of one random variable that is stored in another random variable^47^.

In recent years, the theory of complex networks has been used to study the spatial and temporal evolution of a broad spectrum of complex systems and associated phenomenon from diverse fields such as social networks, transportation networks, communication networks, and networks from computer science and mathematics^48–53^. The application of complex network theory in hydrology and water resources is comparatively new with growing amount of publications on the subjects of connections in rainfall, stream flow, river networks, and virtual water trade networks^54–62^. As for rainfall, Boers *et al*.^63^ used complex network-based concepts to investigate the global pattern of extreme rainfall teleconnections by analyzing the TRMM daily rainfall gridded data. In network theory, clustering coefficient is the standard metric for quantifying the extent to which edges of a network cluster^64^. In order to examine the spatial connections in rain gauge networks, Jha *et al*.^65^ applied clustering coefficient method at six different temporal scales (daily, 2-day, 4-day, 8-day, 16-day, and monthly) using the rainfall data from different rain gauge networks in Australia. They also considered different correlation thresholds to identify the existence of links between stations. Tiwari *et al*.^66^ used complex network theory to reconstruct daily rainfall data and subsequently proposed two variants of Inverse Distance Weighing (IDW) interpolation. Despite the recent extensive use of network theory to study multiple hydrological processes, the suitability of the concept of complex networks to examine the node importance and subsequent gauge prioritization is limited.

In this study we use the complex network-based degree centrality^67^, clustering coefficient^65^, and mutual information^68^ as the parameters to address the node importance quantification in a rain gauge network. In addition, four set of rain gauge selection experiments are introduced to study the influence of the rain gauge density on quantification of node importance. Furthermore, rainfall data at three temporal resolutions, i.e. three-hourly, daily, and monthly are used to study the effect of temporal resolution on the node importance. The specific objectives are: (1) to identify which stations are important and can’t be replaced; (2) to evaluate the rain gauge density and correlation threshold at which the network doesn’t have any links with the neighborhood; and (3) to determine how the importance of rain gauge changes with the temporal resolution. For implementation, we consider the TRMM extracted rainfall data at 692 rain gauge stations, located in the Ganga River Basin in India. To evaluate the performance of the methodology, we use the Soil and Water Assessment Tool (SWAT) hydrological model to predict the stream flow using different rain gauge selection scenarios. We also study the effect of location and elevation of the rain gauges on its importance in overall rain gauge network configuration. To the best of our knowledge the uniqueness of the current study can be highlighted in a number of ways:

- This is the first time the concept of network has been applied to study the rain gauge node importance quantification.
- The comparison of widely used node evaluation parameters i.e. Degree Centrality, Clustering Coefficient, and Mutual Information is reported for the first time.
- The hydrological application in terms of stream flow comparison.
- The use of satellite estimates for rain gauge network evaluation.
- The study area of the current work is Ganga River Basin which is Monsoon dominated, there is hardly any study on the application of Network theory in this region.

## Study Area and Dataset

**Figure 1:**
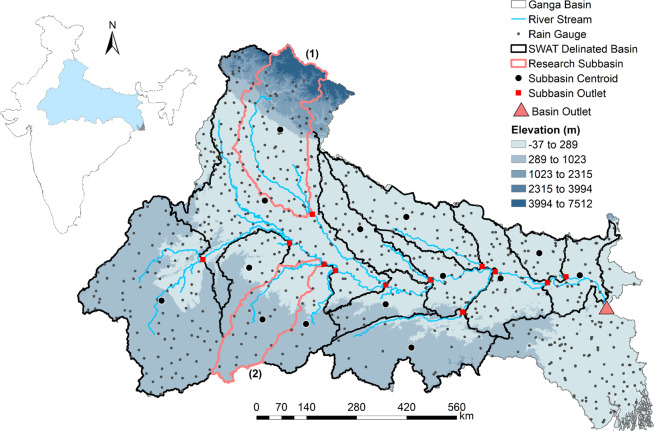
The Ganga River Basin with the location of 692 IMD rain gauges. The dark black lines represent the SWAT delineated basin with 14 subbasins. The figure is generated using the ArcGIS 10.5.1 (https://www.esri.com/en-us/home).

The rainfall data for India’s largest river basin, namely the Ganga River Basin (Fig. 1) is used in this study. The entire Ganga River Basin covers parts of India, Nepal, Bangladesh and China with Indian catchment area of approximately 835744 km^2^ spanning from the latitude 22° 33′ N to 31° 27′ N, and the longitude 73° 23′ E to 89° 06′ E. Within India, it spans the states of Uttarakhand, Uttar Pradesh, Madhya Pradesh (in parts), Bihar, Jharkhand, and West Bengal. The basin is bounded to the north by the Himalayas, to the south by the Vindhyas and Chhotanagpur plateau, to the west by the Aravallis, and to the east by the Brahmaputra hills. The river’s main sources of water are precipitation, subsurface flow, and snow-melt water in the Himalayas. The mean annual rainfall in the basin varies from 300 to 2000 mm. Rainfall is concentrated in the monsoon months from June to September, resulting in low flow rates in the Ganga River and its tributaries during the dry periods of November to April.

The rainfall monitoring network of the Indian Meteorological Department (IMD) plays an important role for hydrometeorological study and coordination of flood mitigation activities. The exact location of all the rain gauges in India is extracted from the IMD Pune data retrieval portal (http://imdpune.gov.in/ndc_new/stations.html). The rain gauges inside the Ganga River Basin is extracted using the shape file of Ganga River Basin (obtained from the National Remote Sensing Centre, Indian Space Research Organization, India). There are 692 IMD rain gauges inside the Ganga River Basin. The elevation of the rain gauges is extracted from the SRTMGL3v003^69^ product (https://lpdaac.usgs.gov/products/srtmgl3v003/). Three hourly, daily and monthly rainfall value at rain gauge stations are extracted using the Tropical Rainfall Measuring Mission^70^ (TRMM) satellite rainfall products. For 3-hourly and monthly temporal scale, TRMM 3B42_V7 and 3B43_V7 rainfall products are downloaded respectively. The daily accumulated (0000 UTC) rainfall is calculated from the 3-hourly rainfall data. The rainfall data at the rain gauge stations are extracted using the Inverse Distance Weighting (IDW) interpolation of the TRMM gridded data (with number of neighbours = 5 and power parameter = 2; as discusses in Tiwari *et al*.^66^).

TRMM satellite data is used instead of IMD rain-gauge data because of unavailability of gauge data at sub daily temporal scale; in addition to that daily IMD gauge data for Ganga River Basin has more than 20 percent gaps, which can influence the analysis significantly. Furthermore, Rain gauge interpolated-gridded data at 1°and 0.25° spatial resolution for more than 100 years exist from IMD. However, the temporal resolution is only daily. In this work, the target applications are: flood forecasting, streamflow prediction, agriculture water demand, and reservoir operation which require subdaily, daily, and monthly rainfall data respectively, hence TRMM satellite products are considered. Only the monsoon season (JJAS) rainfall data from 1/1/1998 to 31/12/2018 is used for the analysis because the major part of the rainfall in Ganga River Basin take place in the monsoon season (The details of monthly average rainfall in the Ganga River Basin are provided in Fig. S1 of supplementary document).

## Methodology

### Network configuration

**Figure 2:**
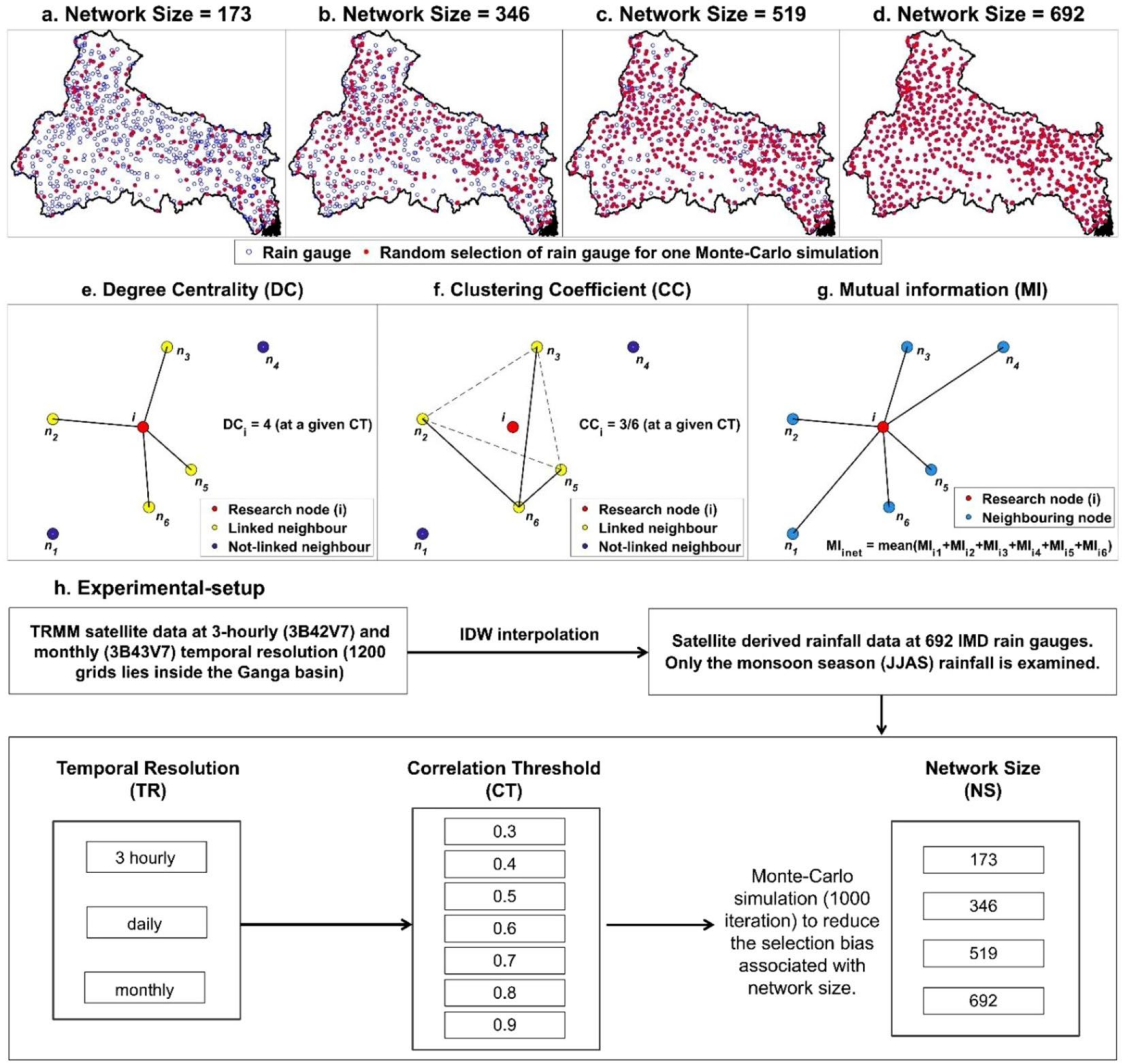
(**a–d**) Random selection of rain gauges with variable Network Size (NS = 176, 314, 519, and 692), 1000 such iterations are computed to reduce the selection bias. Schematic example of network to illustrate the concepts of (**e**) Degree Centrality (DC), (**f**) Clustering Coefficient (CC) and (**g**) Mutual Information (MI). (**h**) Experimental-setup representing variable Temporal Resolution (TR = 3 hours, 1 day, and 1 month), Network Size (NS = 173, 346, 519, and 692) and Correlation Threshold (CT = 0.3 to 0.9). The figure is generated using MATLAB 2017b (https://in.mathworks.com/products/matlab.html).

The fundamental approach to capture the global properties associated with the complex systems is to model them as graph whose nodes represent the dynamical units, and whose links stand for the interactions between them. Mathematically, a graph/network can be represented as G = [P, E], where P is a set of N nodes (P1, P2, …, PN) and E is a set of n links^71^. In the present context, the rain gauge network is considered as the complex network, where rain gauges can be represented as nodes of the network and the connections among them will be the links. We present multiple rain gauge network configuration to study the effect of Rain Gauge Density (RGD) on the rain gauge node importance. Rain gauge network with 25%, 50%, 75%, and 100% of 692 IMD rain gauges are examined. Rain Gauge Density (RGD) below 25% of the present 692 rain gauges is not considered because below 25%, overall network becomes disconnected and almost all rain gauges become important. Rain gauges are selected randomly inside the Ganga River Basin with Network Size (NS) = 173 (25% RGD), 346 (50% RGD), 519 (75% RGD), and 692 (100% RGD) and a network is constructed using the selected rain gauges (Fig. 2(a to d)). For example, in case of NS = 173, there are ^692^C_173_ (more than 10^1000^) ways of selecting 173 rain gauges out of the 692 rain gauges. As the results associated with more than 10^1000^ selections cannot be computed, we randomly select rain gauges (Monte Carlo simulation) with 1000 combinations (to reduce the bias associated with the selection of rain gauges). This way in 1000 iterations, the chances of a particular rain gauge to be selected after 1000 iterations is $\frac{173\times 1000}{692}=250$ times (ideally). When we apply Network theory and calculate node evaluation parameters (i.e. Degree Centrality, Clustering Coefficient, and Mutual Information), we will have nearly 250 values for each node. The results for each rain gauge are presented based on average parameter values. Similarly, results are computed for NS = 344, 519, and 692 rain gauges.

Rainfall data at 3 Temporal Resolutions (i.e. TR = 3 hours, 1 day, and 1 month) is used to study the effect of temporal resolution on node importance quantification. Table 1 represents the climatic and geographic properties of the 4 rain gauge network scenarios presented in Fig. 2(a to d). As shown in 2^nd^ column of Table 1, the RGD increases with NS, which implies the rain network becomes denser with increase in network size. Mean rainfall associated with all the network configurations is equal to the mean rainfall associated with the 692 rain gauges (because in 1000 random selection, almost all the 692 rain gauges are selected equal number of times). The mean and standard deviation of rainfall associated with TR = 3 hours, 1 day, and 1 month is shown in Table 1. Figure 2(h) summarize the overall experimental condition used to study the rain gauge network configurations.

**Table 1:** Climatic and geographic properties of rain gauge network associated with 4 sets of rain gauge network configuration, presented in Fig. 2(a to d). The climatic properties associated with all 692 rain gauges are presented for monsoon (JJAS) season.

Network Size	Rain Gauge Density (rain gauge/km^2^)	Rainfall Statistics (mm)					
		Temporal Resolution					
		3 hours		1 day		1 month	
		Mean	Std.	Mean	Std.	Mean	Std.
NS = 173	20.70 × 10^−5^	0.88	3.23	7.08	15.01	215.95	145.03
NS = 344	41.40 × 10^−5^						
NS = 519	62.10 × 10^−5^						
NS = 692	82.80 × 10^−5^						

### Degree centrality

In a network, we define linked neighbors of node ‘i’ based on the correlation between node ‘i’ and the other nodes in the network. Degree Centrality (DC) is a simple count of the total number of neighbours linked to a node (correlation greater than a Correlation Threshold (CT)), high value of DC implies a node has high number of connected neighbours. As shown in Fig. 2(e), there are 4 linked neigbhour of research node ‘i’ which implies that the DC of node ‘i’ is 4. The DC value for each rain gauge (node) is calculated based on its connection with the remaining 691 rain gauges in the network (without fixing the number of nearest neighbours).

### Clustering coefficient

The clustering coefficient (CC) is a measure of the local density of a network (assigns a score for each node) and quantifies the network’s tendency to cluster^72^. To find the clustering coefficient, the first step is to assign a correlation threshold (CT) to identify the actual neighbours of research node ‘*i*’, i.e. links that have correlations exceeding CT. We refer the number of such neighbours as *k*_*i*_. Then there would be possible $\frac{{k}_{i}({k}_{i}-1)}{2}$ links among *k*_*i*_ neighbours of research node ‘i’. The second step in the estimation of clustering coefficient is to find the possible links between *k*_*i*_ nodes which also exceeds CT. Let *E*_*i*_ be the number of links among neighbouring nodes with correlations exceeding CT, then CC value at node *i* can be mathematically expressed as:

$${\boldsymbol{C}}{{\boldsymbol{C}}}_{{\boldsymbol{i}}}=\frac{2{{\boldsymbol{E}}}_{{\boldsymbol{i}}}}{{{\boldsymbol{k}}}_{{\boldsymbol{i}}}({{\boldsymbol{k}}}_{{\boldsymbol{i}}}-1)}$$

where *E*_*i*_ is the number of links that actually exist between these *k*_*i*_ nodes and (*k*_*i*_ (*k*_*i*_ −1))/2 are the total number of possible links between these *k*_*i*_ nodes. The procedure to find the value of *E*_*i*_ and *k*_*i*_ is repeated for each and every node (research node) in the network to obtain the clustering coefficient associated with each node. Figure 2(f) shows a hypothetical network and illustrates the concept of CC. For example, out of the 6 neighbouring nodes of node *i*, only 4 (yellow dots) have a correlation exceeding the assigned correlation threshold CT. Of the 4 linked neighbouring nodes, there are total 6 possible links (solid and dotted lines). Of the 6 possible links, only 3 have a correlation higher than the assigned CT (only solid lines). Hence for a specified CT, the CC value associated with node *i* is 3/6. The CC value for each rain gauge is calculated based on its connection with the remaining 691 rain gauges in the network (without fixing the number of nearest neighbours).

### Mutual information

Entropy, as defined in the information theory, is a measure of uncertainty of a particular outcome in a random process. The information contained in X can be given by the Shannon entropy^68^ H(X).

$$H(X)=-\,\mathop{\sum }\limits_{k=1}^{K}p({x}_{k}){\log }_{2}[p({x}_{k})]$$

where *k* denotes a discrete data interval, *x*_*k*_ is an outcome corresponding to interval *k*, and *p*(*x*_*k*_) is the probability of *x*_*k*_. The probability *p*(*x*_*k*_) is based on the empirical frequency of variable *X*. The entropy is expressed in bits because the base of the logarithm was assumed to be equal to 2.

Uncertainty of two variables, *X* and *Y*, can be described by the joint entropy *H* (*X*, *Y*).

$$H(X,Y)=-\,\mathop{\sum }\limits_{k=1}^{K}\mathop{\sum }\limits_{l=1}^{L}p({x}_{k},{y}_{l})\log \,[p({x}_{k},{y}_{l})]$$

where *k* denotes a discrete data interval for variable *X*, *l* denotes a discrete data interval for variable *Y*, *p*(*x,y*) is the probability of an outcome corresponding to interval *k* for *X* and interval *l* for *Y*, *K* is the number of class intervals (possible outcomes) for *X*, and *L* is the number of class intervals for *Y*.

Mutual information (MI) is a measure of statistical dependence^73^. The transferable information MI (X, Y) between two rain gauge stations X and Y is the mutual information of X and Y; i.e., the data of station Y can be estimated from the data of station X.

$$MI(X,Y)=H(X)+H(Y)-H(X,Y)$$

where H(X) and H(Y) are the marginal entropy associated with X and Y respectively and H (X, Y) is the joint entropy of X and Y.

In this study, MI is used to identify rain gauge stations with non-repeating information (unique). For each temporal scale, *x*_*k*_ is the rainfall bin with ‘k’ rainfall intervals ranging from minimum to maximum recorded rainfall with a difference of 2.5 mm. The selected bin size of 2.5 mm difference is an ad-hoc decision. Minimum and maximum recorded rainfall is calculated for JJAS season of 1998 to 2018). The Mutual Information associated with a rain gauge ‘i’ in a network with 692 rain gauges is $M{I}_{i}={\sum }_{j=1}^{691}M{I}_{ij}$, where *j* are the rain gauges other than the rain gauge ‘i’ and *MI*_*ij*_ is the mutual information of i and j (as shown in Fig. 2(g)). The MI value for each rain gauge is calculated based on its transferable information with the remaining 691 rain gauges (selecting one at a time) in the network.

### Proxy validation using SWAT hydrological model

This study uses Soil and Water Assessment Tool (SWAT)^74^, a semi-distributed, time-continuous watershed simulator operating on a daily time step, for hydrological modelling. SWAT subdivides a watershed into sub-basins based on topography which are connected by a stream network. Sub-basins are further delineated into Hydrologic Response Units (HRUs), which are defined as land-units with uniform soil, land use, and slope. As the motive here is to study the relative importance of rain gauges in a network and not to predict the streamflow accurately, the model is not calibrated and validated. As shown in Fig. 1, SWAT delineated 14 subbasin in the Ganga River Basin. In order to evaluate the model, the initial three years of the data (1998–2000) served to initialize the model, and the streamflow was calculated for next 18 years (2001–2018). The main goal of the analysis is to verify whether rain gauge selection based on node importance quantification affects the performance of the SWAT model in simulating daily flows. By default, rainfall input data in SWAT are processed by a rather simple, Nearest Neighbour-based method, in which each sub-basin is assigned data from the nearest rain gauge stations to the subbasin’s centroid. In this default method, the simulations results are dependent on very few rain gauges and the data from all rain gauges are not used. We apply spatial interpolation (using Inverse Distance Weighing (IDW) method) of rainfall data and estimate the value of rainfall at the centroid of each subbasin, prior to reading input data in SWAT. We use the IDW method in its standard form (with number of neighbours = 5 and power parameter = 2; as discusses in Tiwari *et al*.^66^) to estimate the rainfall at the centroids of 14 subbasins.

In order to study the implications of node importance quantification based on DC, CC, and MI, we considered only those rain gauges which carry unique information in the network (low value of DC, CC, and MI). For comparison, the rain gauges with redundant information are also considered (high value of DC, CC, and MI). To study the implications of node importance quantifications following nine rain gauge selections based on their importance in a subbasin are introduced:

1. All rain gauges in a subbasin (All_RG)
2. 25 percent of all rain gauges in a subbasin with low DC (Low_DC)
3. 25 percent of all rain gauges in a subbasin with low CC (Low_CC)
4. 25 percent of all rain gauges in a subbasin with low MI (Low_MI)
5. 25 percent of all rain gauges in a subbasin with low DC + CC (Low_DCCC) (i.e. low DC and for comparable DC values (difference of 1), ranking was done based on the associated low CC values).
6. 25 percent of all rain gauges in a subbasin with high DC (High_DC)
7. 25 percent of all rain gauges in a subbasin with high CC (High_CC)
8. 25 percent of all rain gauges in a subbasin with high MI (High_MI)
9. 25 percent of all rain gauges in a subbasin with high DC + CC (High_DCCC) (i.e. high DC and for comparable DC values (difference of 1), ranking was done based on the associated high CC value).

The streamflow at the basin outlet, and subbasin outlets for subbasin 1 and 2 (Fig. 1) are selected for the proxy validation using the hydrologic model. As the model used is not calibrated and validated, normalization of stream flow based on the stream flow generated from the selection of all rain gauge method is done.

$${\rm{N}}{\rm{o}}{\rm{r}}{\rm{m}}{\rm{a}}{\rm{l}}{\rm{i}}{\rm{z}}{\rm{e}}{\rm{d}}\,{\rm{S}}{\rm{t}}{\rm{r}}{\rm{e}}{\rm{a}}{\rm{m}}\,{{\rm{F}}{\rm{l}}{\rm{o}}{\rm{w}}}_{x}=\frac{{{\rm{S}}}_{x}-min({{\rm{S}}}_{All{\rm{\_}}RG})}{max({\rm{S}}{\rm{t}}{\rm{r}}{\rm{e}}{\rm{a}}{\rm{m}}\,{{\rm{F}}{\rm{l}}{\rm{o}}{\rm{w}}}_{All{\rm{\_}}RG})-min({\rm{S}}{\rm{t}}{\rm{r}}{\rm{e}}{\rm{a}}{\rm{m}}\,{{\rm{F}}{\rm{l}}{\rm{o}}{\rm{w}}}_{All{\rm{\_}}RG})}$$

where S represents calculated stream flow associated with a subbasin, All_RG represents input rainfall with all rain gauge selection, x (x = Low DC, Low CC, Low MI, Low DCCC, High DC, High CC, High MI, and High DCCC) represents the input rainfall associated with gauge selection based on node importance quantification. To assess the performance of proposed methodology, following error statistics are used:

#### Absolute Bias Percentage (BP)

measures the tendency of the simulated data to be larger or smaller than their observed counterparts; BP of 0.0 represents accurate model agreement between the observed and simulated values.

$$BP=|{\sum }_{i=1}^{n}\frac{(S(All\_RG)-S(x))\times 100}{S(All\_RG)}|$$

#### Root Mean Square Error (RMSE)

The RMSE^66^ is the expression of the data around the line of best fit. The RMSE does not simply increase with the variance of the errors but increases with the variance of the frequency distribution of error magnitudes. Values equal to zero are optimal, with lower values suggesting good model performance:

$$RMSE=\sqrt{\frac{1}{n}{\sum }_{i=1}^{n}{(S(All\_RG)-S(x))}^{2}}$$

## Results

In this section we present the variation in Degree Centrality (DC) and Clustering Coefficient (CC) with correlation threshold and temporal resolution. Then the relationship of DC and CC with the rain gauge density is discussed. Then the variation of MI with temporal resolution and rain gauge density is discussed. Furthermore, we perform the proxy validation of the node importance quantification methodology. Based on the results of node importance quantification, we present the node importance ranking for 692 IMD rain gauges and 1200 TRMM satellite grids.

### Degree centrality and clustering coefficient

**Figure 3:**
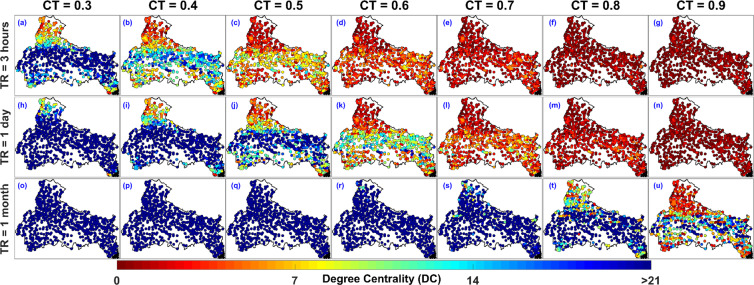
Location of rain gauges in IMD monitored rain gauge network with associated Degree Centrality (DC) value for multiple correlation threshold (CT = 0.3, 0.4, 0.5, 0.6, 0.7, 0.8, and 0.9) and temporal resolution (TR = 3 hours, 1 day, and 1 month). The figure is generated using MATLAB 2017b (https://in.mathworks.com/products/matlab.html).

Degree Centrality (DC) assigns a score based on the number of links held by each node. In the context of node importance quantification, rain gauges with high DC will be the nodes that have high number of connected neighbours (based on a correlation threshold CT). High DC value associated with a rain gauge implies that the data at a rain gauge is not unique (less important node) whereas DC = 0 implies that a node has no connected neighbourhood. Figure 3(a to u) represent the Degree Centrality (DC) values associated with all the 692 rain gauges in the IMD monitored rain gauge network inside the Ganga River Basin.

To visualize the impact of Temporal Resolution (TR) and Correlation Threshold (CT) on the DC values, the DC values are plotted for seven CT (i.e. CT = 0.3, 0.4, 0.5, 0.6, 0.7, 0.8, and 0.9) and three TR (i.e. TR = 3 hours, 1 day, and 1 month), as shown in Fig. 3. For instance, the spatial distribution of DC values for TR = 3 hours and CT = 0.3 (shown in Fig. 3(a)) shows that more than 70 percent of all the rain gauges have DC value greater than 21. Such observation implies that for lower CT values (CT = 0.3 and 0.4), the network is predominantly connected and nodes have high number of linked neighbours. Now, as CT increases from 0.3 to 0.9, the DC values associated with rain gauges decreases and it is really low for CT = 0.8 and 0.9 (see the first row of Fig. 3) implying that the nodes in the network becomes poorly connected for high CT values. Further we kept CT constant as 0.3 and investigated the effect of temporal resolution. By comparing Fig. 3(a,h and o), it can be seen that the DC value increases as TR increases from TR = 3 hours to 1 month. Similarly, for high CT value we observe that the DC value increases as TR increases from 3 hours to 1 month (as shown in Fig. 3(g,n and u) for CT = 0.9), which implies that the nodes in the network becomes more connected for higher TR values.

**Figure 4:**
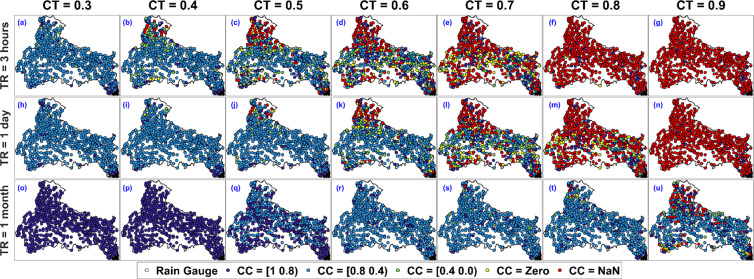
Location of all rain gauges in IMD monitored rain gauge network with associated CC value for correlation threshold (CT = 0.3, 0.4, 0.5, 0.6, 0.7, 0.8, and 0.9) and temporal resolution (TR = 3 hours 1 day, and 1 month). The figure is generated using MATLAB 2017b (https://in.mathworks.com/products/matlab.html).

Next, we discuss the results corresponding to variation in Clustering Coefficient (CC) with CT and TR. When applied to a single node, the clustering coefficient is a measure of the completeness of a node’s neighbourhood. The neighbourhood of a node is well connected or poorly connected with each other if a node has high (close to 1) or low (close to 0) CC value respectively. If CC value becomes NaN, a node will have no or only one connected node in the neighbourhood (DC = 0 and 1). Figure 4(a to u) represent the Clustering Coefficient (CC) values (i.e. CC = NaN, (0.8 1], (0.4 0.8], (0.0 0.4], and Zero) associated with all the 692 rain gauges. The arrangement of plots in Fig. 4 is same as that of Fig. 3. Looking at the effect of CT and TR on CC in Fig. 4, we present only the key observations here. We notice that more than 90 percent of all the rain gauges belongs to CC = [0.8 0.4) for CT = 0.3 and TR = 3 hours. As evident from Fig. 4(a,b), the network is predominantly clustered and the neighbourhood of gauges are well connected for low CT and TR values. Furthermore, for TR = 3 hours, as the CT values increases from 0.3 to 0.9 (see the first row of Fig. 4), the gauges with CC = NaN increases and it is more than 90 percent for CT = 0.9 (as shown in Fig. 4(g)). This indicates that the DC value associated with majority of rain gauges in the network have only 0 or 1 linked neighbour. For constant CT = 0.3 and changing TR, the results show that most of the CC value increases from CC = [0.8 0.4) to CC = [1 0.8), as the TR increases from 3 hours to 1 month (see Fig. 4(a,h and o)). Similarly, as shown in Fig. 4(g,n and u), for CT = 0.9, most of the CC value gets converted to CC = [0.8 0.4) from NaN as TR increases from 3 hours to 1 month, which implies that the network becomes more clustered for higher TR values.

### Effect of rain gauge density on degree centrality and clustering coefficient

**Figure 5:**
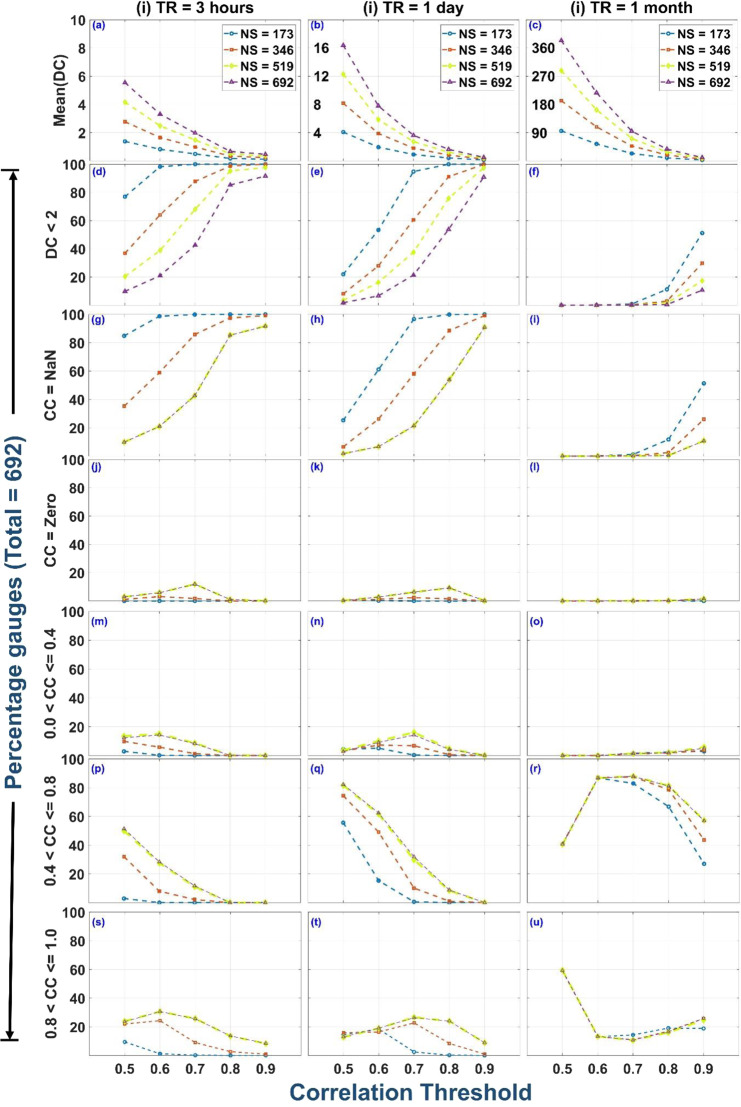
Variation of network properties (i.e. DC and CC) with correlation threshold (CT = 0.5 to 0.9), rain gauge density (Network Size = 173, 346, 519, and 692) and temporal resolution (TR = 3 hours, 1 day, and 1 month). Plots (a-c) show the variation of the mean of Degree Centrality with CT, NS and TR. Plots (d-f) show the percentage of rain gauges with DC < 2. Plots (g-u) show the variation of Clustering Coefficient (CC) range with CT, NS and TR.

We presented the effect of CT and TR on the overall rain gauge network configuration in the previous section. Next, we study the effect of Rain Gauge Density (RGD) on DC and CC values. Rain gauge network with 25%, 50%, 75%, and 100% of 692 IMD rain gauges are examined. Rain gauges are selected randomly with Network Size (NS) = 173 (25% RGD), 346 (50% RGD), 519 (75% RGD), and 692 (100% RGD). To reduce the bias associated with the random selection, Monte Carlo simulation with 1000 iterations is introduced. The results computed from 1000 random selection (Monte Carlo iterations) are averaged. Figure 5(a to c) represent the mean of DC values associated with rain gauges for distinct TR, NS, and CT. Figure 5(a) shows the variation in the mean of DC value with CT ranging from 0.5 to 0.9 for TR = 3 hours (CT < 0.5 is not considered because the network is almost disconnected at lower CT values). The effect of changing NS from 173 to 692 is also included in the Fig. 5. From Fig. 5(a), we can observe that the mean of DC decreases with increase in CT values (0.5 to 0.9) and increases with the increase in NS values (173 to 692). This observation implies that the rain gauge network is poorly connected at high CT values and becomes more connected (less rainfall variability) as the rain gauge density increases (high NS). Now, the effect of increase in TR on the above results can be seen from Fig. 5(a to c). Overall, the mean of DC increases with the increase in TR (notice the limits on the y-axis of Fig. 5(a to c)).

The second row of Fig. 5 shows the variation in percentage of rain gauge with DC < 2 with CT, TR, and NS. A rain gauge with DC less than 2 represents a node with no clustered neighbourhood (for nodes to cluster, at least 3 connected nodes are required). Figure 5(d) shows that the rain gauges with DC less than 2 increases with increase in CT value (0.5 to 0.9) and decreases with the increase in NS value (173 to 692), which implies the rain gauge network is poorly connected and poorly clustered at high CT values and becomes more connected (less rainfall variability) as the rain gauge density increases (high NS).

Figure 5(g to u) represent the variation in CC values with CT, TR, and NS. Figure 5(g to i) quantifies the number of rain gauges with CC = NaN (DC = 0 or 1) for different experimental runs. In the context of node importance quantification, stations with CC = NaN (DC < 2) are most important as they have 0 or 1 linked neighbour, which implies rainfall variability in their neighbourhood is really high. Figure 5(g) shows the percentage rain gauges with CC = NaN for TR = 3 hours. The percentage of rain gauges with CC = NaN increases with increase in CT values and decreases with the increase in NS values, but it is almost equal for NS = 519 (75%), and NS = 692 (100%), which implies the CC values associated with a rain gauge is majorly independent of the rain gauge density for NS > 450. The effect of TR on CC value is quite evident from Fig. 5(g to o). For all CT and NS, the percentage of rain gauges with CC = NaN decreases with increase in TR. All rain gauge networks are subjected to collapse (poorly connected network with more than 50 percent NAN values) with increasing CT values. The Collapse Correlation Threshold (CCT) i.e. CT after which the network becomes predominantly disconnected, varies with the associated temporal resolution. For TR = 3 hours and NS = 692, CCT is 0.6 (For CT greater than 0.6, the number of rain gauges with DC < 2 and CC = NaN increases up to 44 percent of the total rain gauges). For TR = 1 day and NS = 692, CCT is 0.7 and for TR = 1 month and NS = 692, CCT is 0.9. The CCT is important for node importance quantification because for CT = CCT, the network is neither completely connected nor completely disconnected (comparison of node importance is possible), Furthermore CCT defined for rain gauge network in the Ganga River Basin is an ad-hoc decision and the CCT will vary depending upon the associated basin and rainfall characteristics.

As shown in Fig. 5(s to u), the percentage of rain gauges belonging to high CC (i.e. CC range = (0.8 1.0] and DC >  = 2) varies from 5 to 35 percent for TR = 3 hours and 1 day, and from 10 to 60 percent for TR = 1 month. The percentage of rain gauges with CC = 0 are mostly low for all NS and TR (as shown in Fig. 5(j to l)). Nodes with CC = 0 represents the nodes with 2 or more than 2 linked neighbours but neighbours are not connected among themselves. The percentage of rain gauges belonging to low CC (i.e. CC Range = (0 0.4]) are extremely low (nearly less than 10 percent) for all CT and TR values (as shown in Fig. 5(m–o)). The trend of DC and CC values associated with the rain gauges are comparable for TR = 3 hours & 1 day (Column 1 & 2 of Fig. 5) whereas the trend associated with TR = 1 month (Column 3 of Fig. 5) are different from TR = 3 hours & 1 day.

**Figure 6:**
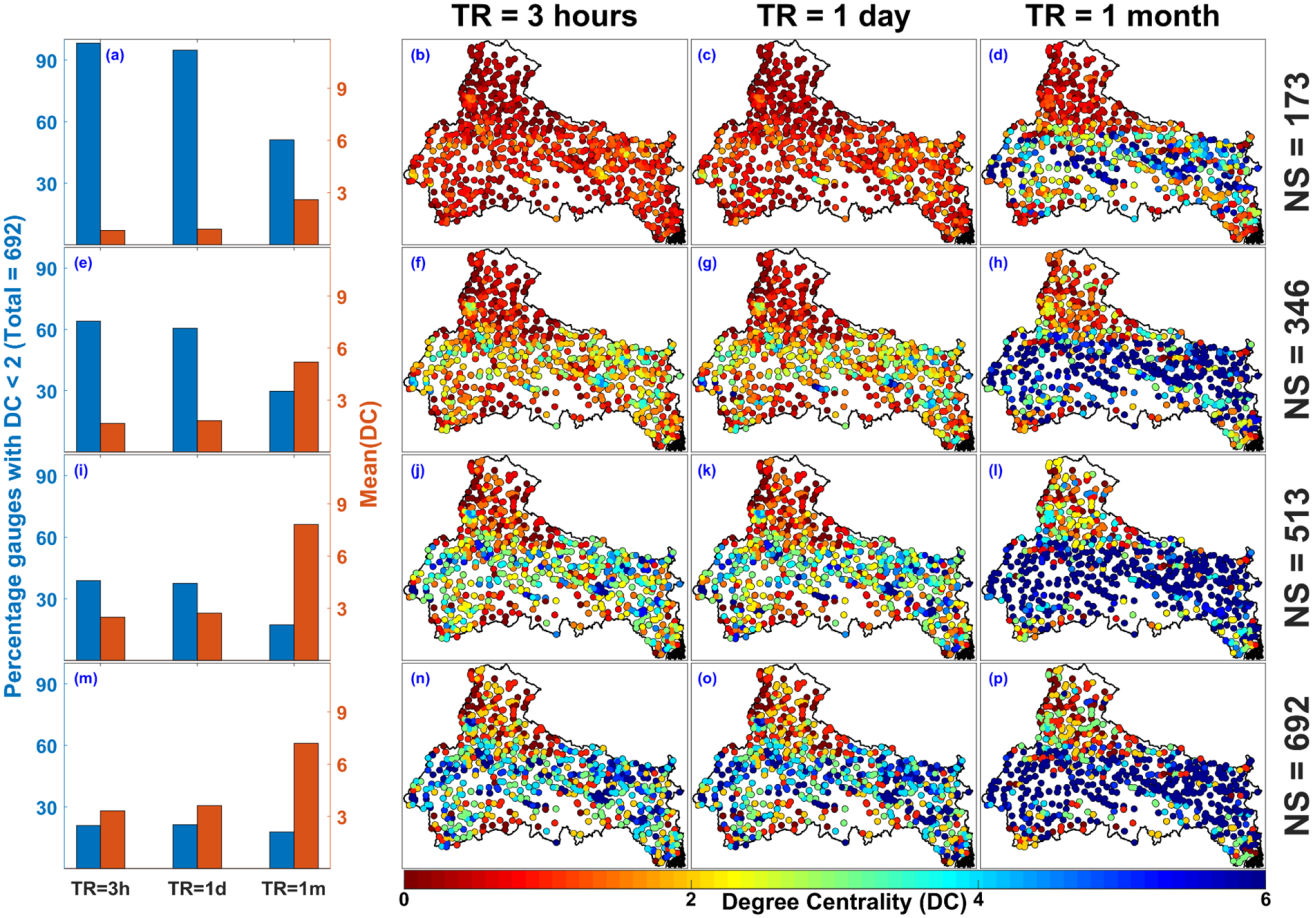
Location of rain gauges with associated Degree Centrality (DC) value for varying network size (NS = 173, 346, 519, and 692) and temporal resolution (TR = 3 hours (CT = 0.6), 1 day (CT = 0.7), and 1 month (CT = 0.9)). The figure is generated using MATLAB 2017b (https://in.mathworks.com/products/matlab.html).

**Figure 7:**
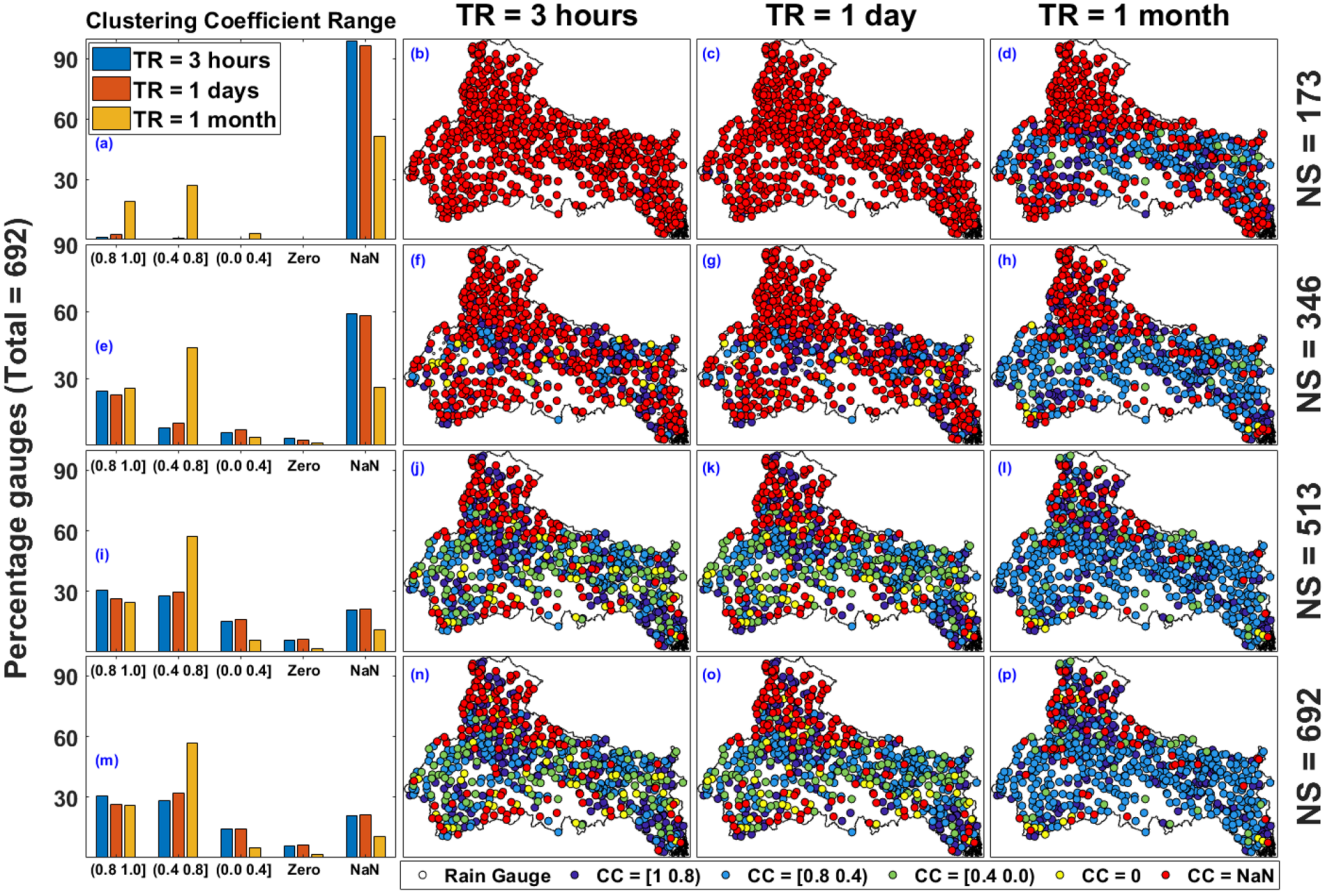
Location of rain gauges with associated Clustering Coefficient (CC) value for varying network size (NS = 173, 346, 519, and 692) and temporal resolution (TR = 3 hours (CT = 0.6), 1 day (CT = 0.7), and 1 month (CT = 0.9)). The figure is generated using MATLAB 2017b (https://in.mathworks.com/products/matlab.html).

To study the spatial distribution of DC and CC values as a function of NS and TR, the DC and CC value associated with all the rain gauges in the proposed network scenarios are plotted in Figs. 6 and 7 respectively. For all the three-temporal resolution, the CT value is considered to be equal to their collapse correlation threshold i.e. CT = 0.6, 0.7 and 0.9 for TR = 3 hours, 1 day and 1 month respectively (as discussed in the previous section). Figure 6(a) shows the statistics associated with DC value for TR = 3 hours, 1 day, 1 month and NS = 173 (25%). For NS = 173, mean of DC is nearly 1 for TR = 3 hours, 1.5 for TR = 1 day and 3 for TR = 1 month. Furthermore, the percentage of rain gauge with DC less than 2 is more than 90 percent for TR = 3 hours and 1 day and it is nearly equal to 50 percent for TR = 1 month (as shown in Fig. 6(a). Similarly, the variation of DC values for different NS values can be estimated from the first column of Fig. 6. The spatial distribution of rain gauges with associated DC values is shown in second, third and fourth column of Fig. 6 for TR = 3 hours, 1 day, and 1 month respectively. For NS = 150 and TR = 3 hours and 1 day, DC value associated with all the rain gauges is close to 1 (as shown in Fig. 6(b,c)), which implies DC values are mostly low for a network configuration with low rain gauge density. For NS = 173 and TR = 1 month, rain gauges with low DC values are mainly situated on the periphery of the network configuration (as shown in Fig. 6(d)), which implies DC values are mostly low for a network configuration with low density. As the network size increases, the rain gauge density increases and subsequently gauges acquire higher DC value (as shown in Fig. 6(n–p)).

Figure 7 represent the spatial distribution of CC values associated with distinct NS and TR. First column of Fig. 7 represents the quantification of spatial distribution of rain gauges. Figure 7(a) shows the percentage of rain gauges in different CC ranges for TR = 3 hours, 1 day and 1 month and NS = 173. For NS = 173, NaN values are more than 80 percent in case of TR = 3 hours and 1 day, while for TR = 1 month, NaN values are nearly equal to 40 percent (as shown in Fig. 7(a)). Similarly, quantification of rain gauges in different CC ranges for distinct NS can be estimated from the first column of Fig. 7. The spatial distribution of rain gauges with associated CC values is shown in second, third and fourth column of Fig. 7 for TR = 3 hours, 1 day, and 1 month respectively. For NS = 173 and TR = 3 hours and 1 day, CC value associated with nearly all the rain gauges is equal to NaN (as shown in Fig. 7(b,c)), which implies that the CC values are mostly NaN for a network configuration with low rain gauge density. For NS = 173 and TR = 1 month, NaN values are nearly equal to 40 percent of NS and are mainly situated on the periphery of the network configuration (as shown in Fig. 7(d)). As network size increases, the network density increases and subsequently rain gauges acquire high CC value and there are smaller number of NaN values near the centre for NS = 692 (as shown in Fig. 7(n–p)). Rain gauges with CC = zero are mainly situated inside the network configuration and not on the periphery, mainly because CC = zero implies a node have connections with its neighbourhood but neighbours are not connected among themselves. The distribution of CC values is almost similar in case of TR = 3 hours and 1 day (as shown in the 2nd and 3rd column of Fig. 7). Overall, we observe that the location of rain gauges with CC = NaN are predominantly located in the northern most part of the Ganga River Basin, which is the high elevated Himalayan region (as shown Fig. 7).

### Mutual Information

**Figure 8:**
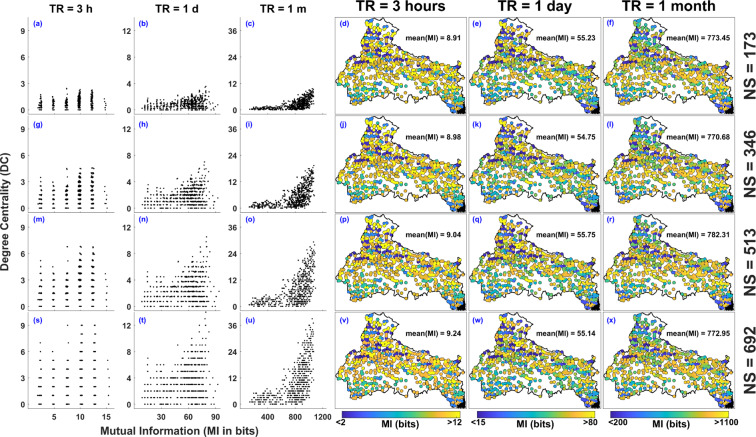
Relationship between Degree Centrality (DC) and Mutual Information (MI in bits) along with spatial distribution of MI associated with rain gauge network configuration for NS = 179, 344, 519, and 692 and TR = 3 hours, 1 day, and 1 month. The figure is generated using MATLAB 2017b (https://in.mathworks.com/products/matlab.html).

Mutual Information (MI) is one of the widely used parameters for rain gauge network evaluation. The underlying basis for examining networks based on the concept of entropy is that the stations should have as small MI as possible, meaning that the stations should be independent from each other. Therefore, the results are perceived as follows: Lower value of MI implies that the stations share less common information and hence are considered to be more independent. Whereas larger MI value shows that the stations are mostly duplicating the same information. To understand the relationship between Degree Centrality and Mutual Information, the comparison of DC and MI values associated with all the rain gauges in the proposed experimental setups (see Fig. 2(g)) are plotted in Fig. 8. The comparison of DC and MI values associated with NS = 173 and TR = 3 hours is shown in Fig. 8(a). The MI value varies from 2 to 15 bits and DC from 0 to 2.8 for all the rain gauges in the network. As shown in Fig. 8(a to c), the DC and MI values associated with NS = 173, increases with increase in TR (please note the changing limits of x- and y-axis of plots). Figure 8(a,g,m and s) shows the variation of MI and DC value associated with TR = 3 hours and NS = 173, 344, 519 and 692 respectively. The variation of MI with NS is not significant whereas the DC value increases with the increase in NS, which implies the variation in MI values with rain gauge density is not significant. The 4^th^, 5^th^ and 6^th^ column of Fig. 8 shows the spatial distribution of MI values along with the mean of MI values. Overall, the variation in the MI with Network Size (rain gauge density) is not really large but it varies significantly with TR. Furthermore, the rain gauges located in the low elevated planner regions (near the centre of basin) have high MI values whereas most of the rain gauges in high elevated Himalayan region have low MI values.

### Proxy validation of node importance quantification using hydrologic modeling

In this section we present the results of using rainfall input based on node importance quantification to a SWAT model. From Section 3.5, recall that we are considering 14 subbasins in Ganga River basin. We consider daily rainfall data from 1^st^ Jan 1998 to 31^st^ December 2018 for setting up SWAT model. It is worth pointing out that SWAT considers the nearest rain gauge to the subbasin’s centroid as the input rainfall data. Even though we have rain gauges spread over the entire catchment, to avoid randomness in data selection and also to incorporate the application of node importance quantification we have interpolated the rainfall value at the centroid of each subbasin. First, to calculate rainfall value at subbasin centroid, IDW interpolation is applied to all the rain gauges (All_RG) present inside a subbasin. The generated rainfall and simulated stream flow from All_RG rain gauge selection is considered as the reference/baseline for the subsequent output rainfall and streamflow generated from the selection of the specific rain gauges based on the node importance quantification. Eight rain gauge selection methodlogy, namely, Low_DC, Low_CC, Low MI, Low_DCCC, High_DC, High_CC, High_MI, and High_DCCC are used to select only 25 percent of the total rain gauges in a subbasin. In DCCC, we consider both DC and CC value in selecting a set of rain gauges. For Low_DCCC, the selection of rain gauges is based on low DC value, but for rain gauges with comparable DC value, the selection of rain gauges is based on low CC value. Similarly for High_DCCC, the selection of rain gauges is based on high value of DC and CC.

The RMSE and BP are used as the criteria to analyze the deviation of the input rainfall and simulated stream flow by 8 node importance quantification methodologies in comparision to rain gauge selection based on all rain gauges (All_RG). Table 2 represents the error statistics (RMSE and BP) for (i) calculation of rainfall at the centroid of 14 subbasin (mean error), (ii) normalized stream flow at subbasin outlet 1 (Himalayan Region), (iii) normalized stream flow at subbasin outlet 2 (Betwa), and (iv) normalized streamflow at Ganga Basin outlet (Fig. 1).

**Table 2:** Error statistics associated with computed (a). rainfall at subbasin centroid, (b). streamflow at subbasin 1 outlet, (c). streamflow at subbasin 2 outlet, and (d). streamflow at basin outlet, errors associated with different gauge selected were calculated by considering rainfall/streamflow output computed from selection of all rain gauges as the base parameter.

Rain gauge selection criteria	Root Mean Square Error (RMSE)				Absolute Bias Percentage (BP)			
	Rainfall at the centroid of all 14 subbasins	Stream flow at the outlet of subbasin 1	Stream flow at the outlet of subbasin 2	Stream flow at the basin outlet	Rainfall at the centroid of all 14 subbasins	Stream flow at the outlet of subbasin 1	Stream flow at the outlet of subbasin 2	Stream flow at the basin outlet
Low_CS	3.956	0.047	0.080	0.032	59.723	22.132	42.68	14.173
Low_CC	3.927	0.069	0.080	0.031	59.035	26.261	42.68	13.473
Low_MI	4.142	0.110	0.100	0.140	65.882	86.418	86.381	69.623
Low_CSCC	3.576	0.054	0.062	0.029	56.359	19.917	34.608	12.348
High_CS	4.174	0.076	0.110	0.071	59.967	25.904	52.445	21.903
High_CC	4.132	0.120	0.120	0.046	60.986	61.246	64.628	16.562
High_MI	4.972	0.170	0.100	0.150	73.209	107.74	65.11	69.869
High_CSCC	4.003	0.072	0.110	0.069	57.530	23.697	52.666	21.641

As shown in Table 2, for all the simulations the output errors (RMSE and BP) associated with the rain gauge selection based on Low_DCCC (fourth row of Table 2) have lowest error, mainly because the rain gauges with Low_DCCC have unique information (nodes with low number of linked neighbours and poor connection among the neighbours), hence small number of rain gauges (only 25 percent of all the rain gauges) with Low_DCCC can represent the properties associated with the overall basin quite efficiently. The error associated with rain gauge selection based on Low_DC and Low_CC (first and second row of Table 2) have also comparable results, mainly because rain gauges with low DC(DC < 2) have low CC (CC = NaN) values (in most cases). The errors associated with selection based on Low_MI and High_MI (third and seventh row of Table 2) is significantly high in comparison to the associated error with selection based on DC and CC. The high error associated with the selection based on MI implies that the mutual information may not be the efficient parameter for node importance quantification. Furthermore, among the stream flow simulated at all the 3 outlets i.e. Subbasin 1 (Himalaya), Subbasin 2 (Betwa) and Basin outlet (Ganga), the error associated with the Ganga Basin outlet has the least errors whereas the error associated with Betwa Basin outlet is the highest.

**Figure 9:**
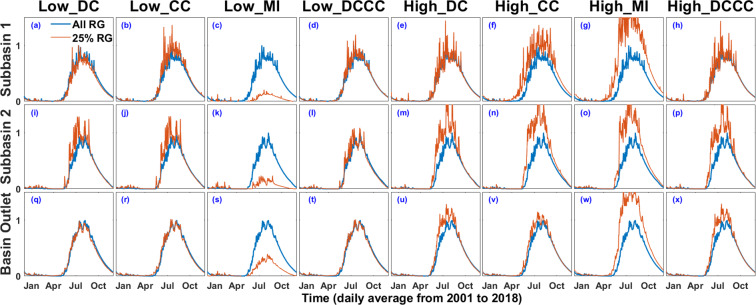
Time Series plot of SWAT simulated stream flow (normalised) at the outlet of (a). Subbasin 1 (Himalaya) (b). Subbasin 2 (Betwa) (c). Basin (Ganga River Basin).

Figure 9(a to x) represent the time series plot of daily stream flow computed from SWAT hydrological model, recorded at the outlet of subbasin 1 (Himalaya), subbasin 2 (Betwa) and Ganga River Basin (Fig. 1). The stream flow is simulated using 9 rain gauge selection experiments, which includes all rain gauge selection (All_RG), selection of gauges with Low_DC, Low_CC, Low_MI, Low_DCCC, High_DC, High_CC, High_MI, and High_DCCC. Average of the stream flow simulated from 01/01/2001 to 31/12/2018 is shown in Fig. 9. Figure 9(a) shows the daily simulated stream flow from the rain gauge selection based on All_RG (blue) and Low_DC (orange) for subbasin 1 in Himalayan region. It is clear from the visual inspection of Fig. 9(a) that the stream flow generated from Low_DC is quite close to the stream flow generated from the All_RG selection. Overall, the stream flow simulated from Low_DC and Low_CC selection are really close to the stream flow output from All_RG selection for all the 3 outlets i.e. Himalayan, Betwa, and Ganga River Basin outlet (as shown in Fig. 9(a,b,i,j,q and r)). Furthermore, the streamflow generated from Low_DCCC is closest to All_RG in comparison to other node quantification methodologies at all the 3 outlets (as shown in Fig. 9(d,l, and t).

The streamflow from Low_MI selection is an underestimation of All_RG selection (as shown in Fig. 9(c,k and s)and the stream flow generated from High_MI gauge selection is an overestimation of the stream flow generated from All_RG slection (as shown in Fig. 9(g,o and w). The overall results suggest that the DC and CC based methods are more efficient than MI based methods for the purpose of node importance quantification. The relationship of yearly rainfall variation with node importance quantification based on Low_DCCC are shown in Fig. S3 and S4 of the supplementary document.

### Node Importance Quantification

**Figure 10:**
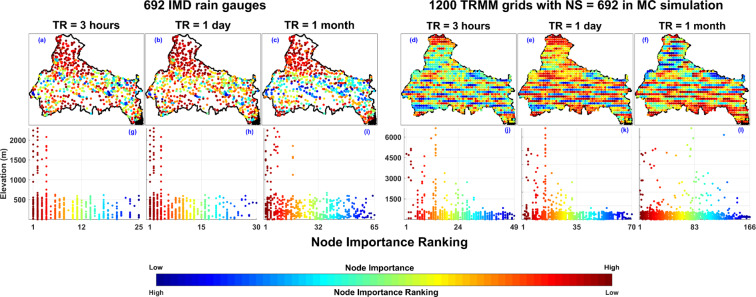
Node importance quantification (based on DCCC) for IMD rain gauge network with 692 nodes (**a–c**) and for 1200 TRMM satellite grids with NS = 692 in 1000 iterations of Monte Carlo simulation (**d–f**). Plots from g to l represents the relationship of rain gauge importance with its elevation (elevation of the rain gauges is extracted from SRTMGL3v003^69^ product). The figure is generated using MATLAB 2017b (https://in.mathworks.com/products/matlab.html).

Based on the results discussed in the previous section, node importance quantification using DCCC method is presented in Fig. 10. The DCMC method represents the node importance quantification based on the low DC value and for comparable DC value the importance quantification is based on the low CC value. The DC and CC values are calculated for the Collapse Correlation Threshold (CCT) associated with each temporal resolution (CCT = 0.6, 0.7, and 0.9 for TR = 3 hours, 1 day, and 1 month respectively). High node importance implies that the rain gauge contains unique information and cannot be replaced. Figure 10(a to c) show the spatial distribution of node importance ranking of 692 rain gauges for TR = 3 hours, 1 day, and 1 month (low node importance ranking implies high node importance). For TR = 3 hours, the importance ranking of all the 692 rain gauges in the IMD network is presented in Fig. 10(a and g). Rain gauges with similar DC and CC values have the equal importance ranking in the network. As the distribution of DC and CC varies with the TR, range of node importance ranking also vary with the TR. The presented results show the relative significance of each rain gauge for specific temporal resolution. The results cannot be compared between the temporal resolution because CCT varies with TR. From the visual inspection of Fig. 10(a to c), it is evident that the rain gauges situated at the high elevated Himalayan region are relatively more important whereas the rain gauges situated in the planner region near the center of basin are relatively less important. In addition, Fig. 10(g to i) show the relationship between the elevation of 692 rain gauges and their importance in the network. As shown in Fig. 10(g to i), rain gauges situated at high elevation are most important.

Further, to suggest modification in the present IMD rain gauge network and to present an optimum rain gauge network with network size = 692, 1000 random selection of 692 grids out of the 1200 TRMM grids are simulated. The results associated with 1000 iterations are averaged to get a value for each grid. Figure 10(d to f) represent the importance quantification of 1200 TRMM grids inside the Ganga River Basin for NS = 692 in Monte Carlo simulation. For TR = 3 hours and 1 day the result shows that the grids in the Himalayan regions are mostly important whereas for TR = 1 month the high elevated Himalayan regions do not have many grids of high importance, which implies the variability at monthly scale is not high in the Himalayan region (as shown in Fig. 10(d to f)). Furthermore, grids situated at high elevation are most important in case of TR = 3 hours and 1 day, but the relationship between grid importance and their elevation is not conclusive in case of TR = 1 month.

## Conclusion and Discussion

The node importance quantification of a rain gauge network is important for various hydrological applications. In this paper, we develop complex network-based, node importance quantification methodologies to identify the most important rain gauges in different rain gauge network configurations. To understand the dependency of rainfall variability on network size and temporal resolution we proposed and studied multiple experimental runs. We also investigated the characteristics of network properties in terms of degree centrality, clustering coefficient and mutual Information. We compared the proposed network configurations to study the effect of network density on the importance of specific rain gauges in the network. The main results of this study can be summarized as follows:

- The Collapse Correlation Threshold (CCT) is an important parameter to evaluate any rain gauge network. Our results show that the CCT strongly depends on the temporal resolution of recorded data. For TR = 3 hours, 1 day, and 1 month, we found that the CCT is 0.6, 0.7, and 0.9 respectively (as shown in Fig. 5).
- For a rain gauge network with network density lower than 20 × 10^−5^ rain gauges/km^2^, almost all the rain gauges in the network are critical (as shown in Table 1, Figs. 6 and 7).
- The elevation of rain gauges strongly affects their significance in a network configuration. Rain gauges at higher elevation are more important in recording the rainfall variability (as shown in Fig. 10).
- The location of a rain gauge strongly influences its importance in a network. The rain gauges situated at the periphery of the network are mostly important in recording rainfall variability (as shown in Fig. 10).
- The mutual information associated with a rain gauge network configuration is almost independent of rain gauge density and network size but strongly depends on the temporal resolution of the recorded data (as shown in Fig. 8).
- Degree Centrality and Clustering Coefficient are important parameters for node importance quantification whereas the results obtained by choosing Mutual Information as the quantification parameter are not conclusive (as shown in Table 2 and Fig. 9).
- Updating (removal or installation) the present IMD rain gauge network based on the node importance map presented in Fig. 10 can help in achieving optimum rain gauge network in the Ganga River Basin.
- The TRMM 3B42_V7 (3-hourly) and 3B43_V7 (monthly) rainfall data at 25 km spatial resolution are used to perform the present study. The IMERG rainfall product at higher temporal (30 minute) and spatial (~10 km) resolution is also available from 2000 to 2019. The effect of selection of TRMM or IMERG data on rain gauge importance quantification is presented in the Fig. S3 and Table S1 of the supplementary material.
- The encouraging results for the quantification of node importance in this study seem to indicate that the approach has the potential to address problems related to extreme rainfall forecasting, changing rainfall patterns and filling gaps in spatial data. Based on the objective, the node importance quantification can be designed for observation data at adequate spatial and temporal resolution.
- This complex network theory-based technique can be further used for node importance quantification in the study of various spatially distributed parameters.

## Supplementary information

Supplementary information.

## Data Availability

Three-hourly (3B42_TRMM_V7) and Monthly TRMM (3B43_TRMM_V7) rainfall data used in this study are available online at https://pmm.nasa.gov/data-access/downloads/trmm. The location of all the IMD monitored rain gauges in India is available online at http://imdpune.gov.in/ndc_new/stations.html. The elevation of rain gauges is extracted using SRTMGL3v003 product (https://lpdaac.usgs.gov/products/srtmgl3v003/).
