# Supplementary material for: Quantification of node importance in rain gauge network: influence of temporal resolution and rain gauge density
Source: Sci Rep. 2020 Jun 17;10:9761. doi: 10.1038/s41598-020-66363-5 (PMC7300113; doi:10.1038/s41598-020-66363-5)
Supplement: Supplementary file 1 — Supplementary information. [file 41598_2020_66363_MOESM1_ESM.docx]

**Supplementary Material**

**Quantification of node importance in rain gauge network: influence of temporal resolution and rain gauge density**

Shubham Tiwari^1^, Sanjeev Kumar Jha^1*^, Ankit Singh^1^

^1^Indian Institute of Science Education and Research Bhopal, Madhya Pradesh, India

^*^Corresponding Author

Email: [sanjeevj@iiserb.ac.in](mailto:sanjeevj@iiserb.ac.in)

**Monthly variation of rainfall in Ganga River Basin**

In this study, we use the concept of the complex networks to evaluate the Indian Meteorological Department (IMD) monitored 692 rain gauge in the Ganga River Basin. Three hourly, daily and monthly rainfall value at rain gauge stations are extracted using the Tropical Rainfall Measuring Mission (TRMM) satellite rainfall products. Only the monsoon season (JJAS) rainfall data from 1/1/1998 to 31/12/2018 is used for the analysis because the major part of the rainfall in Ganga River Basin take place in the monsoon season (as shown in Figure S1).


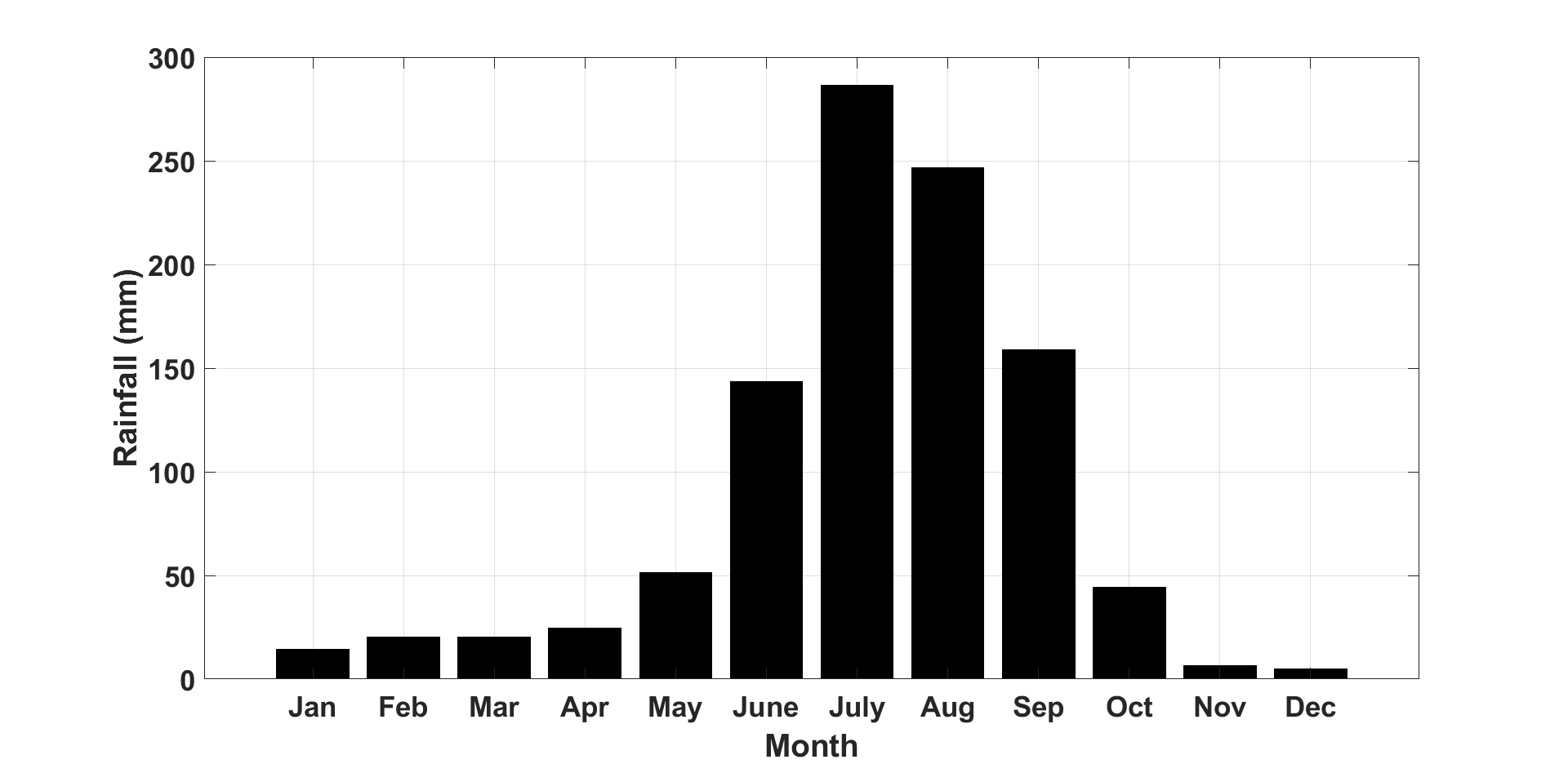


**Figure S1.** Monthly average rainfall inside the Ganga River Basin (Extracted using TRMM product from 01/01/1998 to 31/12/2018)

**Dependency of presented rain gauge importance quantification on the selection of satellite rainfall product (TRMM/IMERG)**

The TRMM 3B42_V7 (3-hourly) and 3B43_V7 (monthly) rainfall data at 25 km spatial resolution are used to perform the present study. The IMERG rainfall product at higher temporal (30 minute) and spatial (~10 km) resolution is also available from 2000 to 2019. To explore the relationship of presented node importance quantification with the satellite rainfall product, the TRMM and IMERG satellite rainfall products inside the Ganga is compared. We observed that the TRMM grid density (1200 grids inside the Ganga River Basin) is comparable with the present IMD rain gauge density (692 rain gauges inside the Ganga River Basin). The IMERG satellite data has 7500 grids inside the Ganga River Basin. Hence it can be used to design a much denser rain gauge network. In terms of temporal scales, IMERG data provides rainfall at 30-minute interval which will be again a valuable information for studying rain gauge network at a very high temporal resolution. However, to use rainfall data at 30-minute temporal scale (from IMERG) for a rain gauge network design in Ganga River Basin will be completely an unrealistic study, which is beyond the present and near future capability of IMD. The total monsoon season (JJAS) rainfall obtained from TRMM and IMERG satellite products are comparable for each year from 2000 to 2019 (as evident from Figure S2).

Furthermore, to study the dependency of rain gauge importance quantification on the selection of satellite rainfall product (TRMM/IMERG), we extracted the rainfall value at 692 rain gauges using TRMM and IMERG satellite products. Subsequently, we estimated the Root mean square error (RMSE) and Bias percentage (BP) associated with IMERG data with respect to TRMM at 3 hourly, daily and monthly temporal scales. We noticed that the RMSE and BP values are low (Table S1). Hence in the context of present IMD rain gauge network, we observe that the rain gauge importance quantification will not be significantly affected with the selection of TRMM or IMERG data.


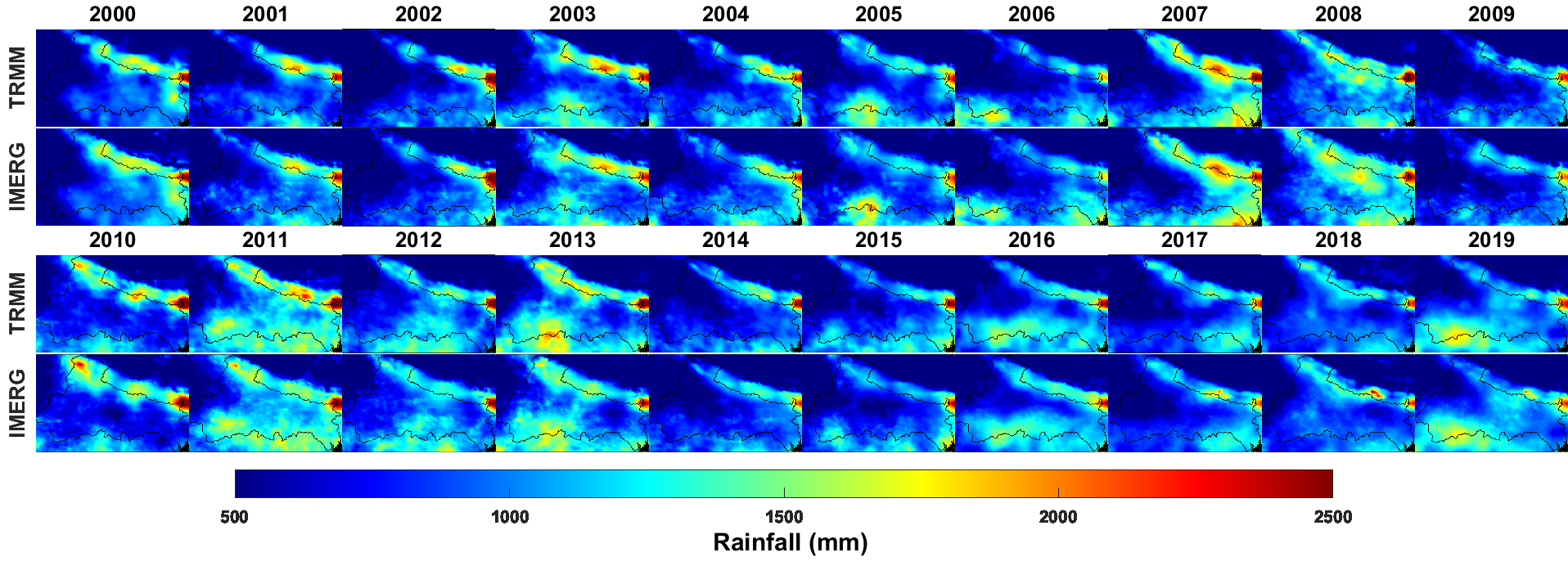
**Figure S2.** Comparison of TRMM and IMERG satellite rainfall products in Ganga River Basin. The plots represent the total monsoon season (JJAS) rainfall. The figure is generated using MATLAB 2017b (<https://in.mathworks.com/products/matlab.html>).

**Table S1.** The error statistics associated with IMERG rainfall data with respect to TRMM rainfall data (extracted at 692 IMD rain gauge stations inside the Ganga River Basin).

| **Temporal Resolution** | **RMSE** | **BP** |
| --- | --- | --- |
| 3 hours | 5.464 | 1.0249 |
| 1 day | 2.689 | 2.082 |
| 1 month | 2.690 | 10.508 |

**Role of yearly rainfall variations on rain gauge importance quantification**

Installation and maintenance of rain gauge networks requires sufficient funding and adequate institutional framework. As updating a rain gauge network on yearly basis is not possible because of the associated cost and labour, the rain gauge networks are generally designed using the rainfall records of several years. But the yearly rainfall variation can significantly affect the performance of a rain gauge network. In Figure S3, we plot daily rainfall data averaged over the Ganga River Basin from year 1/1/2000 to 31/12/2018. As expected, rainfall does show large variation yearly; no two consecutive years have similar rainfall pattern. The effect of yearly rainfall variation on the presented rain gauge importance quantification methodology can be seen by the streamflow generated from the SWAT model. Figure S3 shows that the streamflow (normalized) generated using two rain gauge selection scenarios: (a) all rain gauges in the network (All_RG) and (b) 25% rain gauges with low degree centrality and clustering coefficient (Low_DCCC). As evident from Figures S3, the fluctuation in streamflow generated from Low_DCCC in comparison to that generated from All_RG is mainly observed during the high intensity rainfall events (which mostly occurs during JJAS).

Figure S4 represents the quantification of the errors associated with streamflow generated from Low_DCCC with respect to streamflow generated from All_RG. The relationship of the associated errors with the yearly rainfall variations is also shown in Figure S4. The visual inspection of Figure S4 suggest that when the annual rainfall is about 1000mm, the RMSE and BP values are relatively low. However, when there is low or very high annual rainfall, the error is generally high. Overall, we can say that the node importance quantification accurately represents spatio-temporal variability of rainfall during the average rainfall years whereas the representation is relatively less accurate during those years with relatively higher or lower annual rainfall.


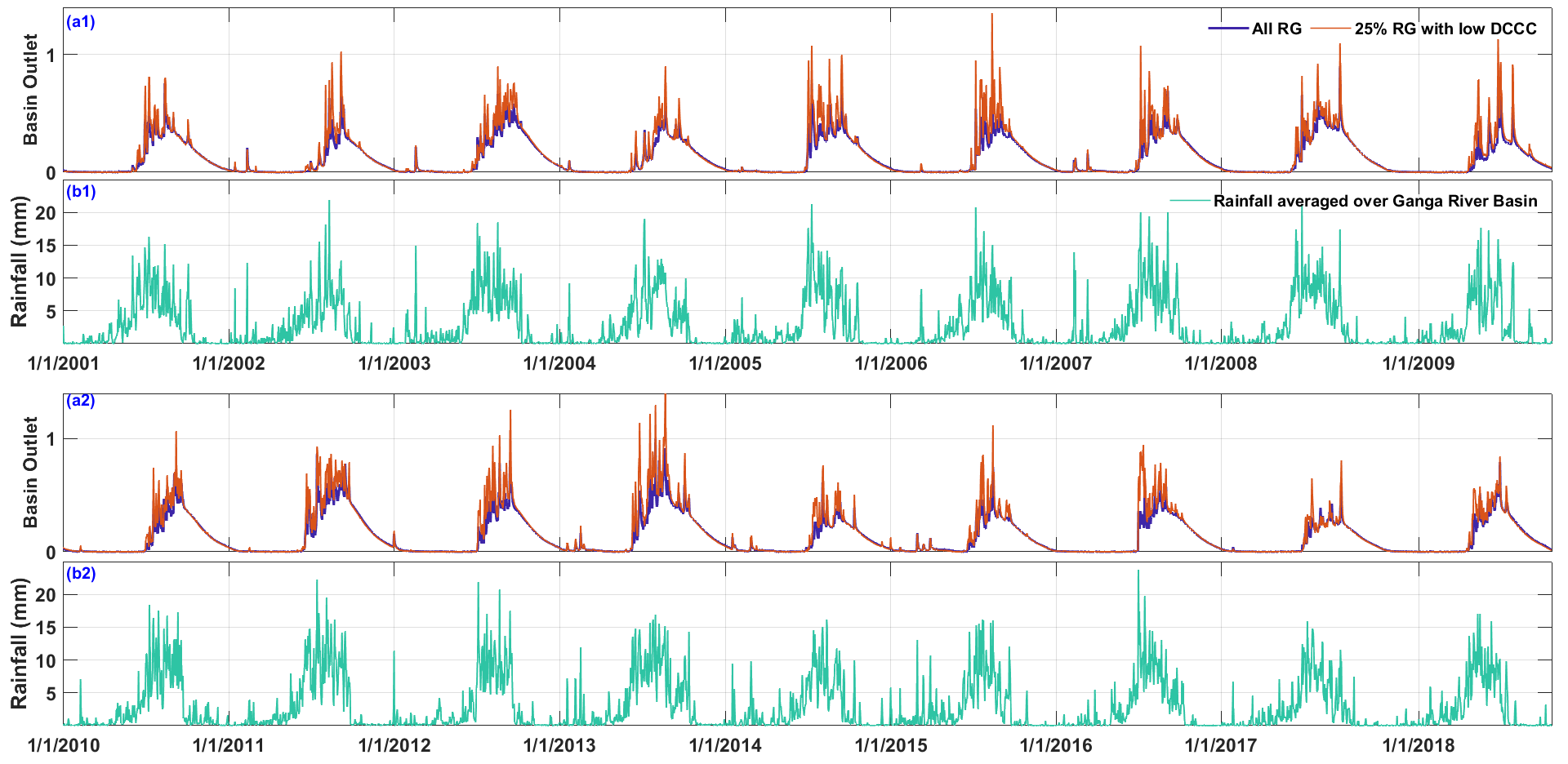


**Figure S3.** Time Series plot of SWAT simulated stream flow (normalised) at the outlet of Ganga River Basin.


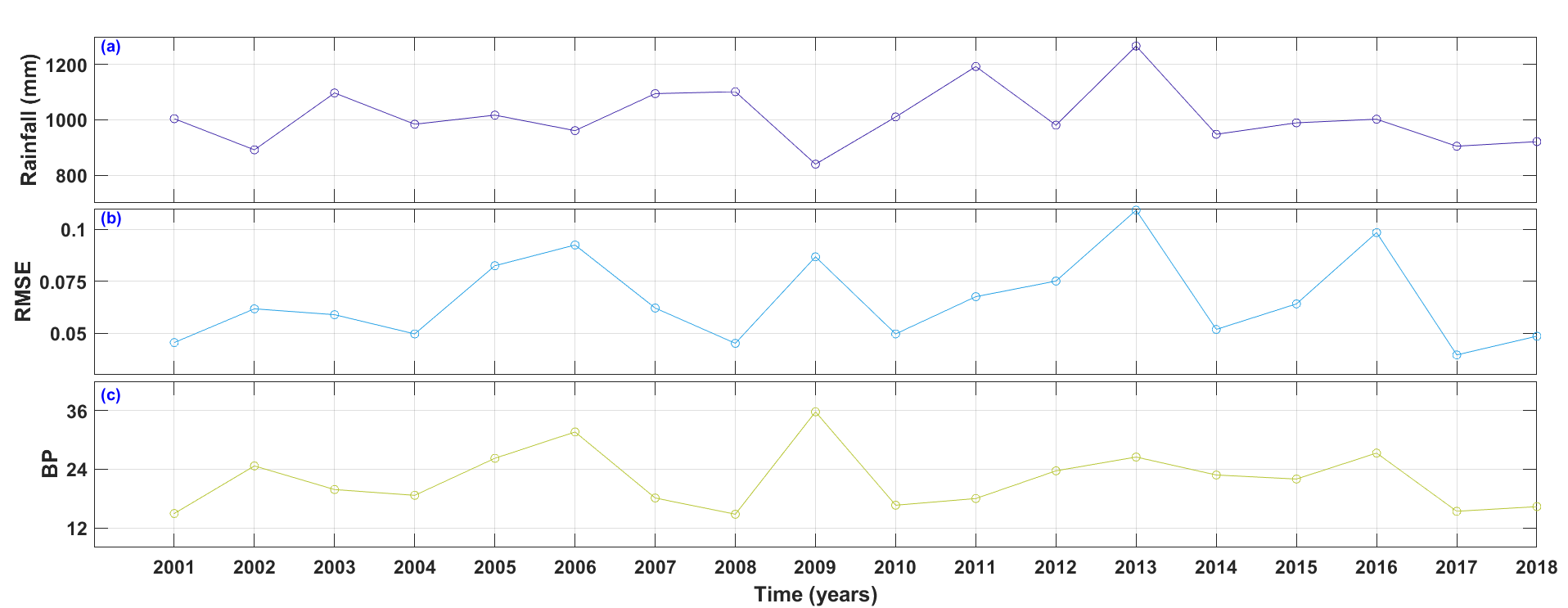


**Figure S4.** The (a). mean Rainfall inside the Ganga River Basin, (b). RMSE and (c). BP. The RMSE and BP are associated with stream flow generated from 25 percent of rain gauges with respect to the stream flow generated from all rain gauge selection.
